# Specific gene-regulation networks during the pre-implantation development of the pig embryo as revealed by deep sequencing

**DOI:** 10.1186/1471-2164-15-4

**Published:** 2014-01-03

**Authors:** Suying Cao, Jianyong Han, Jun Wu, Qiuyan Li, Shichao Liu, Wei Zhang, Yangli Pei, Xiaoan Ruan, Zhonghua Liu, Xumin Wang, Bing Lim, Ning Li

**Affiliations:** State Key Laboratories for Agrobiotechnology, College of Biological Sciences, China Agricultural University, Beijing, China; Animal Science and Technology College, Beijing University of Agriculture, Beijing, China; Novogene Bioinformatics Institute, Beijing, China; College of Life Science, Northeast Agricultural University, Haerbin, China; Stem Cell and Developmental Biology, Genome Institute of Singapore, Singapore, Singapore; Beijing Institute of Genomics, Chinese academy of Sciences, Beijing, China

## Abstract

**Background:**

Because few studies exist to describe the unique molecular network regulation behind pig pre-implantation embryonic development (PED), genetic engineering in the pig embryo is limited. Also, this lack of research has hindered derivation and application of porcine embryonic stem cells and porcine induced pluripotent stem cells (iPSCs).

**Results:**

We identified and analyzed the genome wide transcriptomes of pig *in vivo*-derived and somatic cell nuclear transferred (SCNT) as well as mouse *in vivo*-derived pre-implantation embryos at different stages using mRNA deep sequencing. Comparison of the pig embryonic transcriptomes with those of mouse and human pre-implantation embryos revealed unique gene expression patterns during pig PED. Pig zygotic genome activation was confirmed to occur at the 4-cell stage via genome-wide gene expression analysis. This activation was delayed to the 8-cell stage in SCNT embryos. Specific gene expression analysis of the putative inner cell mass (ICM) and the trophectoderm (TE) revealed that pig and mouse pre-implantation embryos share regulatory networks during the first lineage segregation and primitive endoderm differentiation, but not during ectoderm commitment. Also, fatty acid metabolism appears to be a unique characteristic of pig pre-implantation embryonic development. In addition, the global gene expression patterns in the pig SCNT embryos were different from those in *in vivo*-derived pig embryos.

**Conclusions:**

Our results provide a resource for pluripotent stem cell engineering and for understanding pig development.

**Electronic supplementary material:**

The online version of this article (doi:10.1186/1471-2164-15-4) contains supplementary material, which is available to authorized users.

## Background

Embryonic stem cells (ESCs) can differentiate into any cell type of the three germ layers as well as into male and female germ cells [1, 2]. As such, they offer great potential for regenerative medicine and animal breeding. ESCs have been derived from the mouse, rat and humans, but only mouse ESCs have been tested in tetraploid complementation assays [3–6]. The derivation of such cell lines later helped researchers understand the molecular mechanisms governing pluripotency and early embryonic cell fate commitment. However, because of the short lifespan of the mouse, mouse models are insufficient for evaluating the long-term effects of cell replacement or cell therapy. On the other hand, the *in vivo* developmental potential of ESCs cannot be directly tested in humans. Pigs are an ideal model for preclinical development and design of therapeutic approaches because their organs are morphologically and functionally similar to humans [7, 8]. For this reason, pig ES cell lines must be generated with the same *in vivo* developmental potential as mouse ES cells. Since the 1990s, attempts have been made to derive pig ESCs and the generation of porcine iPSCs has been recently reported. However, during this time, no stable porcine cell lines have been capable of germ-line transmission; thus, they are not authentically pluripotent [9–12]. These problems may be due to inadequacies in the currently used *in vitro* culture conditions that cannot support pluripotency maintenance. This is partly a result of a lack of information regarding unique molecular mechanisms of early pig embryonic development [8, 13].

The progression from fertilization to implantation among mammals is highly conserved, and the morphologic stages are similar [14]. Few interspecies differences do occur, such as time spent at each stage, timing of zygotic genome activation (ZGA) and cell lineage commitment initiation. Gene-regulation networks of mouse PEDs have been extensively studied and reported [15–17], but scarce information regarding molecular mechanism of pig early embryonic development as well as other large domestic animals has limited our knowledge of developmental biology and aspects of engineering their stem cells.

Genome-wide transcriptome analysis may reveal unique gene regulation networks during PED that would be useful in the biological studies of undifferentiated ESCs and pre-implantation embryos [18, 19]. Transcriptome analysis of early pig embryos may also elucidate differentiation characteristics of putative porcine ESCs and iPSCs to optimize *in vitro* culture conditions for the generation of true pig ESCs. Therefore, we compared early pig *in vivo* fertilized-derived and mouse *in vivo* fertilized-derived embryo transcriptomes and mapped a putative gene regulation network during pig PED. This work represents a significant step towards characterizing normal and cloned pig early embryos using genome-wide gene expression patterns.

## Results

### Dynamic gene expression landscapes of PED

**Figure 1:**
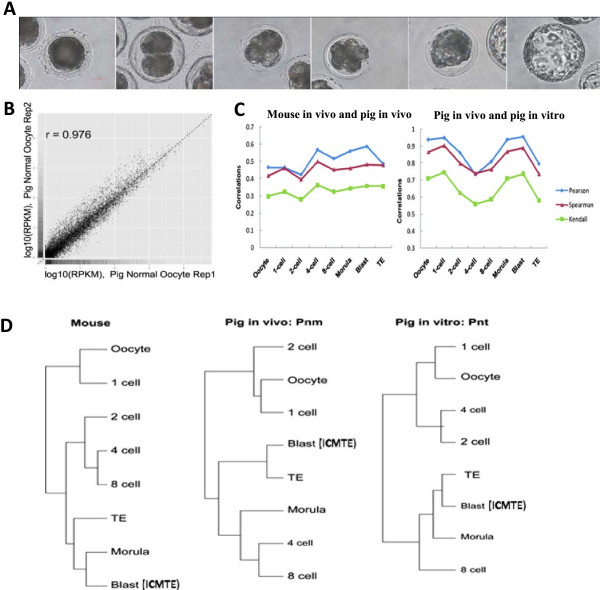
**Gene expression landscape of pre-implantation embryonic development. (A)** The porcine pre-implantation embryonic developmental process (from left to right: oocytes, 1-cell, 2-cell, 4-cell, 8-cell, morula and blastocyst embryos). **(B)** Representative Pearson correlation coefficient for replicates of pig oocyte samples calculated using log2-based RPKM. **(C)** Correlation analysis of the gene expression at corresponding time points of mouse and pig pre-implantation embryos derived *in vivo* or pig pre-implantation embryos derived *in vivo* and *in vitro*. Three methods were used to estimate the similarity between expressed patterns. **(D)** Unsupervised hierarchical clustering of the gene expression profiles of mouse and pig pre-implantation embryos. Read counts were used as the input for average agglomerative clustering analysis via Euclidean distance measurement.

To identify the gene regulation networks that act during *in vivo* pig PED, we isolated mRNA for deep sequencing from *in vivo* porcine (Pnm) and mouse embryos (Ms) and porcine SCNT embryos (Pnt) harvested at different stages during PED (Additional file 1: Table S1 and Figure 1A). At the blastocyst stage, we physically separated the blastocyst into two parts, one containing pure trophectoderm (TE) and the other (ICMTE) containing the inner cell mass (ICM) and part of the TE (Additional file 2: Figure S1). The protocol for small cell number mRNA-sequencing was optimized to identify and analyze pig and mouse genome-wide transcriptomes at various stages of early development [20]. To verify the sequencing data reproducibility, we collected two sets of pig *in vivo* embryos at each stage (Additional file 1: Table S1). The Pearson correlation coefficient for replicates calculated by log_10_ RPKM ranged from 0.865 to 0.985, indicating reliable sequencing data. Representative results observed in oocyte samples are shown in Figure 1B. To further validate the data and analysis methods, we performed three correlation measurements to estimate the transcriptome similarity between mouse and pig embryos derived *in vivo* and between *in vivo* pig embryos and pig SCNT embryos (Figure 1C). All three correlation coefficient calculations revealed similar patterns at different stages with no obvious differences between mouse and pig embryos from 4-cell stage. However, a major difference was found at the 4-cell stage between the pig SCNT-derived and *in vivo* fertilized-derived pig embryos. Also a prominent difference existed between TE cells at the blastocyst stage (Figure 1C and Additional file 3: Table S2).

To explore the relationships between different developmental stages, unsupervised hierarchical clustering analysis was used to evaluate similarities in global gene expression patterns. Data showed that the biggest difference between consecutive time points during mouse PED occurred from the 1- to 2-cell stage, and the second biggest difference occurred from the 8-cell to morula stages. A similar pattern was observed in normal pig embryos but the major difference occurred between the 2-cell and 4-cell stages (Figure 1D and Additional file 3: Table S2). In addition, the morula clustered together with the ICMTE (Figure 1D) in mice, while the ICMTE clustered with the TE in pigs. In the human, a different clustering pattern exists (Additional file 2: Figure S2A) [21]. Thus pig, mouse and human PED have different patterns of gene expression.

An obvious difference was observed in the hierarchical order between pig *in vivo* derived embryos and pig SCNT embryos (Figure 1D; Additional file 2: Figure S2B). In SCNT embryos, the largest difference existed from the 4- to 8-cell stage and the next largest difference was noted from the morula to blastocyst stage (Figure 1D, Additional file 3: Table S2). Thus the events of reprogramming that occurred after reconstruction of SCNT embryos changed the gene expression patterns during their pre-implantation development.

### Comparative analysis of the gene regulation networks important to maternal deposition and zygotic gene activation between pigs and mice

**Figure 2:**
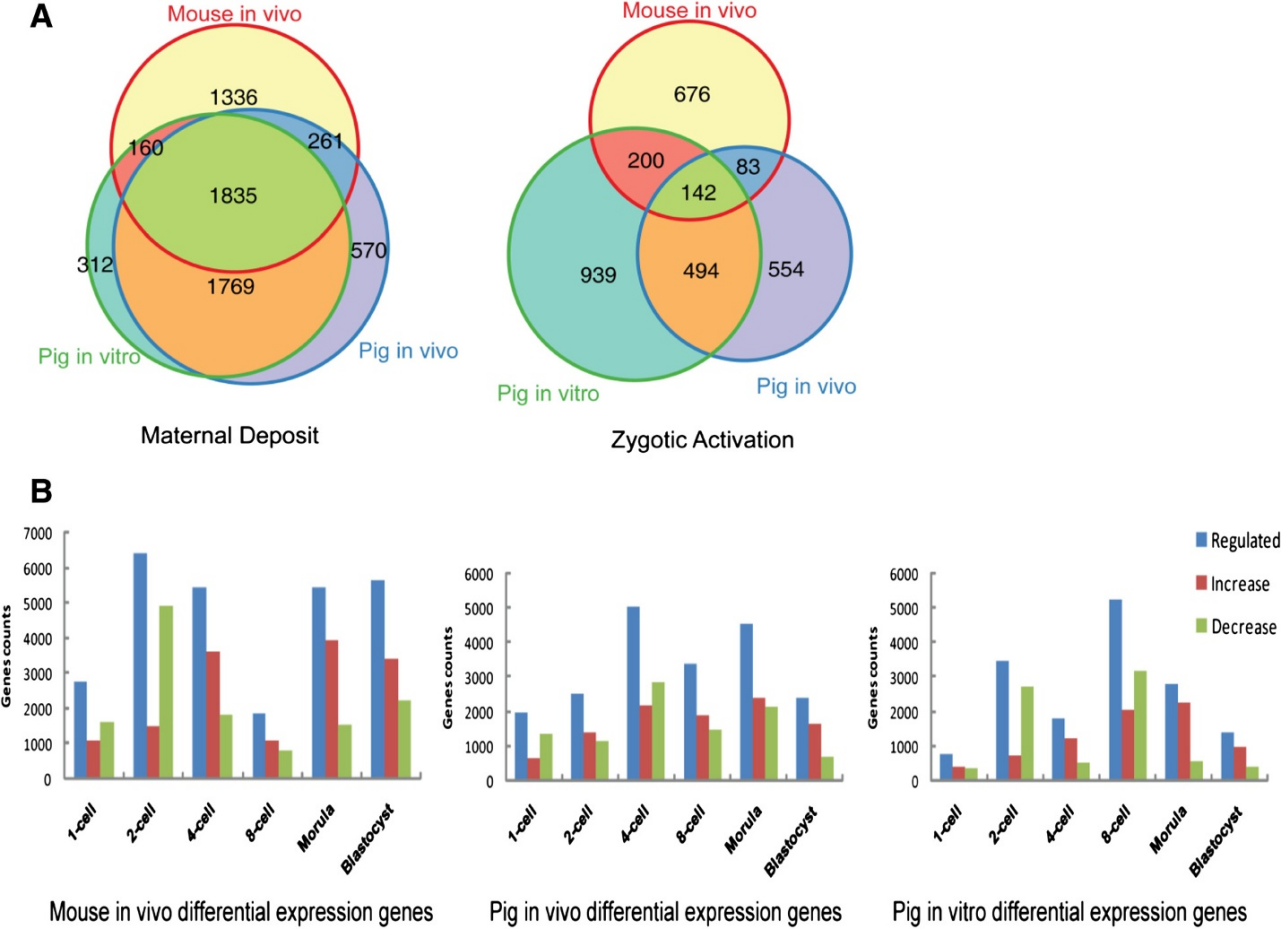
**Identification of maternal deposition and zygotic activation. (A)** Venn diagrams of maternal deposit (left) and zygotic activation (right) conserved across mice and pigs or specific to mice or pigs. Maternal deposit genes are defined by RPKM > 3 in oocyte samples. **(B)** Histograms of regulated genes (sum of increased and decreased genes). The expression of regulated genes was found to be significantly different between two joint time points when corrected (*P*-value <0.05 and fold change > 2).

After fertilization, there is a transition from maternal to zygotic developmental control which requires both degradation of maternal RNAs and zygotic genome activation (ZGA) [22]. We first identified maternal transcripts from oocyte transcriptomes and zygotic activated transcripts in mice and pigs based on transcription trends (Figure 2; Additional file 4: Table S3) [14]. *In vivo* fertilized-derived pig embryos shared 81.3% of their maternal mRNA with *in vitro* SCNT-derived embryos, and 47.3% of transcripts were shared with mouse embryos (Figure 2A left). *In vivo* pig embryos shared 17.7% of their zygotic activation transcripts with those in mice embryos, and 50.0% were shared in SCNT-derived embryos (Figure 2A right). These results suggest that porcine maternal transcripts are more conserved than pig zygotic activated transcripts, a finding that is consistent with previous reports on humans and cattle embryos [14]. Maternal transcript specialization may explain the low efficiency of interspecies animal cloning [23–27]. These data also suggest that transcriptional factors in the ooplasm can more effectively identify donor-cell-specific DNA domains of the same species.

To evaluate the differences in zygotic activation between mice and pigs, we counted the number of up- and down-regulated genes at different stages of early cleavage development. Major bursts of transcription occurred at the 2-cell stage in mice, at the 4-cell stage in normal pig embryos, and at the 8-cell stage in pig SCNT embryos (Figure 2B). These results may correspond to the zygotic activation stage [22]. The timing of ZGA varies among species and occurs between 4- and 8-cell stages in humans and reportedly between 1- and 2-cell stages in mice [16, 28]. Based on our transcriptome analysis, porcine ZGA occurs at 4-cell stage, consistent with previous reports [29]. ZGA appeared to be delayed by one cell cycle in pig SCNT embryos, compared with *in vivo* normal embryos (Figure 2B). It is associated with the transcripts during SCNT embryonic development that might result from artificial micromanipulation [30]. For example, reconstructed embryos undergo only active demethylation at a slow and gradual pace, whereas normal embryos exhibit both active and passive demethylation at faster rates [31]. We found that genes associated with demethylation, such as Dnmt3b and Dnmt1, changed their expression level after demethylation in both normal and SCNT embryos (Additional file 2: Figure S3).

Transition from maternal to zygotic transcripts is accompanied by expression of specific genes whose products associate with protein binding. Genes of known ontology (GO:0005515, protein binding) were used to construct heat-maps of hierarchical clustering (Additional file 2: Figure S4A), showing that the protein binding-associated transcripts were enriched in mouse 2-cell embryos, pig normal 4-cell stage and morula embryos, and pig 8-cell SCNT embryos. This supports the concept that ZGA timing events differ in mice and pigs (Additional file 2: Figure S4B). Also, mouse and pig embryos showed differences in transcripts associated with ATP-synthesis-coupled-proton transportation. The column dendrogram was reordered to show the ATP synthesis genes are differentially expressed in the two species. ATP-synthesis-coupled-proton transports were highly expressed at the 4-cell stage in normal pig embryos, the 8-cell stage in pig SCNT embryos, and the 2-cell stage in mouse embryos, which supports the previous findings that ZGA events occur at these stages (Additional file 2: Figure S4C). Genes and GO analysis data regarding maternal deposit and ZGA are shown in Additional file 5: Table S4.

### Comparative analysis of gene regulation in pig and mouse during the three committed lineages of embryonic development

Two lineage segregation events occur in mammalian PED. In mice, the first event occurs at E3.5, when ICM and TE formation are mediated by Pou5f1 (also known as Oct4). The second event is regulated by Nanog and Gata6, resulting in the segregation of the primitive endoderm and epiblast lineages from the ICM at the blastocyst stage [32]. To evaluate differences in the regulation of lineage segregation between pig and mouse embryos, we first examined gene expression in pig ICM cells.

### Profile identification of putative pig ICM transcripts

**Figure 3:**
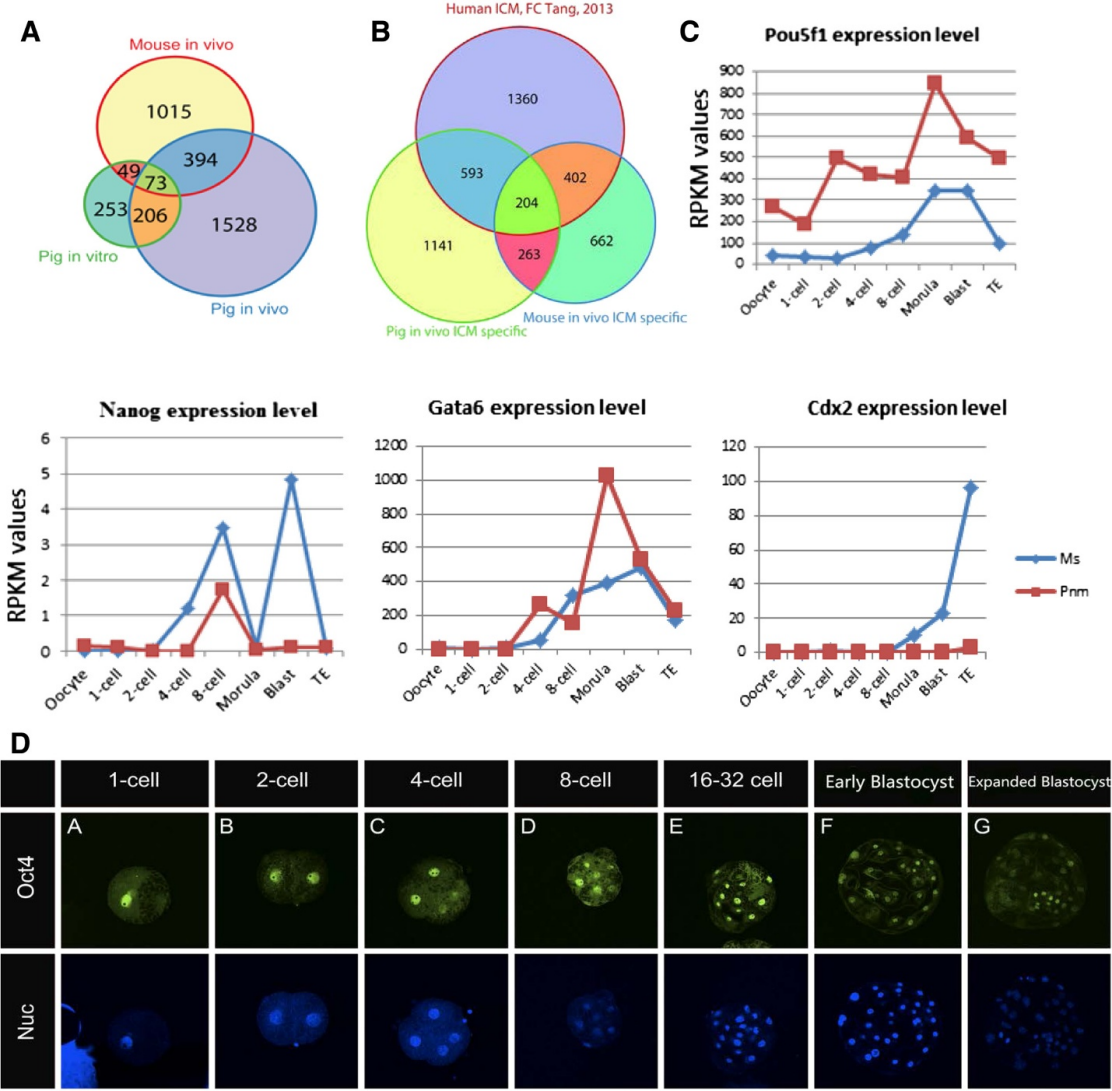
**Gene expression profiling in putative pig ICM cells. (A)** Venn diagram of ICM-specific gene expression of mouse and pig embryos derived *in vivo* and pig embryos derived *in vitro*. ICM-specific genes were found to be expressed at higher levels than genes expressed in the blastocyst of the TE sample when corrected for *P*-value <0.05, fold change >2, and Blast RPKM >3. **(B)** Venn diagram of genes expressed in the mouse, pig and human ICM [1]. **(C)** Pou5f1, Nanog, Gata6 and Cdx2 expression levels were measured using RPKM values and RNA-sequencing results. **(D)** Pou5f1 immunostaining at different stages of porcine PED.

We split the porcine blastocyst into two parts using an ultra-sharp blade. One half contained the TE only and the other comprised both ICM and TE cells (ICMTE). We then identified specific genes expressed in porcine ICM cells by comparing transcriptomes of pure TE and ICMTE in pig normal embryos. To validate this method, we identified 1,531 ICM-specific genes through differential expression analysis between ICMTE and TE mRNA sequencing data in mouse embryos (Figure 3A). 74.9% of the ICM-specific genes (Additional file 2: Figure S5A) and 71.5% of the ICMTE-specific genes (Additional file 2: Figure S5B) were included in ICM datasets that have been previously reported [18]. This suggests that our data is reliable (Figure 3A; Additional file 5: Table S4). With this method, we identified 2,201 putative porcine ICM-specific genes in *in vivo*-derived embryos and 581 genes in SCNT embryos from the first sample set (Figure 3A). Similar results were obtained with the second set of normal pig embryos. With these, 2,559 human ICM-specific genes were identified [21]. We found that 30.5% of the putative mouse ICM-specific genes and 35.3% of the human ICM-specific genes are shared with pig *in vivo*-derived embryos (Figure 3B and Additional file 2: Figure S5C), and that these contained ES cell markers such as Pou5f1, Tbx3 and Gata6. (Additional file 5: Table S4). GO analysis results suggested that mouse and pig ICM cells share pathways including those within the cell cycle, cell division, *in utero* embryonic development, the TGF-beta receptor signaling pathway, and positive regulation of transcription. However, pig ICM-specific genes were clustered with unique pathways, such as fatty acid metabolism, heat shock protein binding, and fatty acid beta-oxidation, which may be important in pig early embryonic development (Additional file 2: Figure S5B; Additional file 5: Table S4). Many ICM-specific genes in the mouse embryos were associated with mouse ES cell pluripotency. Typical mES cell markers such as Pou5f1, Sox2, Esrrb, Klf4, Mest and Tbx3 were expressed in the putative ICM, and these are believed to act within the pathways involved with stem cell maintenance and response to retinoic acid (Additional file 5: Table S4).

To determine whether the same key regulation factors act in the same signaling pathways in both pig and mouse embryos during PED, we analyzed signaling pathway patterns relevant to development or pluripotency: the TGF-beta, MAPK, Jak-Stat and Wnt pathway. Interestingly, different genes within the same pathways were activated in mouse and in pig embryos. Acvr2b, Id1 and Amhr2 were highly expressed in mouse ICM in the TGF-beta pathway, whereas Smad1, Smurf1 and Id4 were highly expressed in pig ICM. Likewise, differences were also observed for the Jak-Stat, MAPK and Wnt signaling pathways (Additional file 2: Figure S7). These data indicate that unique regulatory signaling pathways may be associated with porcine ICM development, and that these are different during lineage segregation between mouse and pig embryos.

### Analysis of molecular markers for lineage commitment during PED

Next, we analyzed the expression of genes that govern early lineage segregation, specifically Oct4, Cdx2, Nanog, Sox2 and Gata6, in putative ICM and TE cells. Consistent with previous reports, we found that Oct4 expression was restricted to the ICM at the blastocyst stage during mouse PED (Additional file 2: Figure S8A). This was consistent with analysis of deep sequencing data (Figure 3C). The pattern of Oct4 expression in *in vivo* pig embryos was similar to that of mouse embryos (Figure 3C,D). These results suggest that Oct4, which regulates early development in mice, may have a similar function for lineage segregation in pigs. In mice, the expression of Cdx2 increased gradually from the 8-cell stage, and was predominant in TE at the blastocyst stage (Figure 3C). We observed a similar tendency of Cdx2 expression during pig PED, but the expression level was much lower than in mice (Figure 3C; Additional file 2: Figure S8B). Nanog expression was very low and could not be detected in the morula or blastocyst by immunostaining during pig PED (Figure 3C; Additional file 2: Figure S8C). In addition, the pattern of Gata6 and Sox2 expression in pig PED was different to that observed in mice (Figure 3C; Additional file 2: Figure S8D,E). This suggests that the regulation of second lineage segregation events during PED might differ between mice and pigs.

### Comparison of gene regulation in lineage segregation during pig and mouse PED

**Figure 4:**
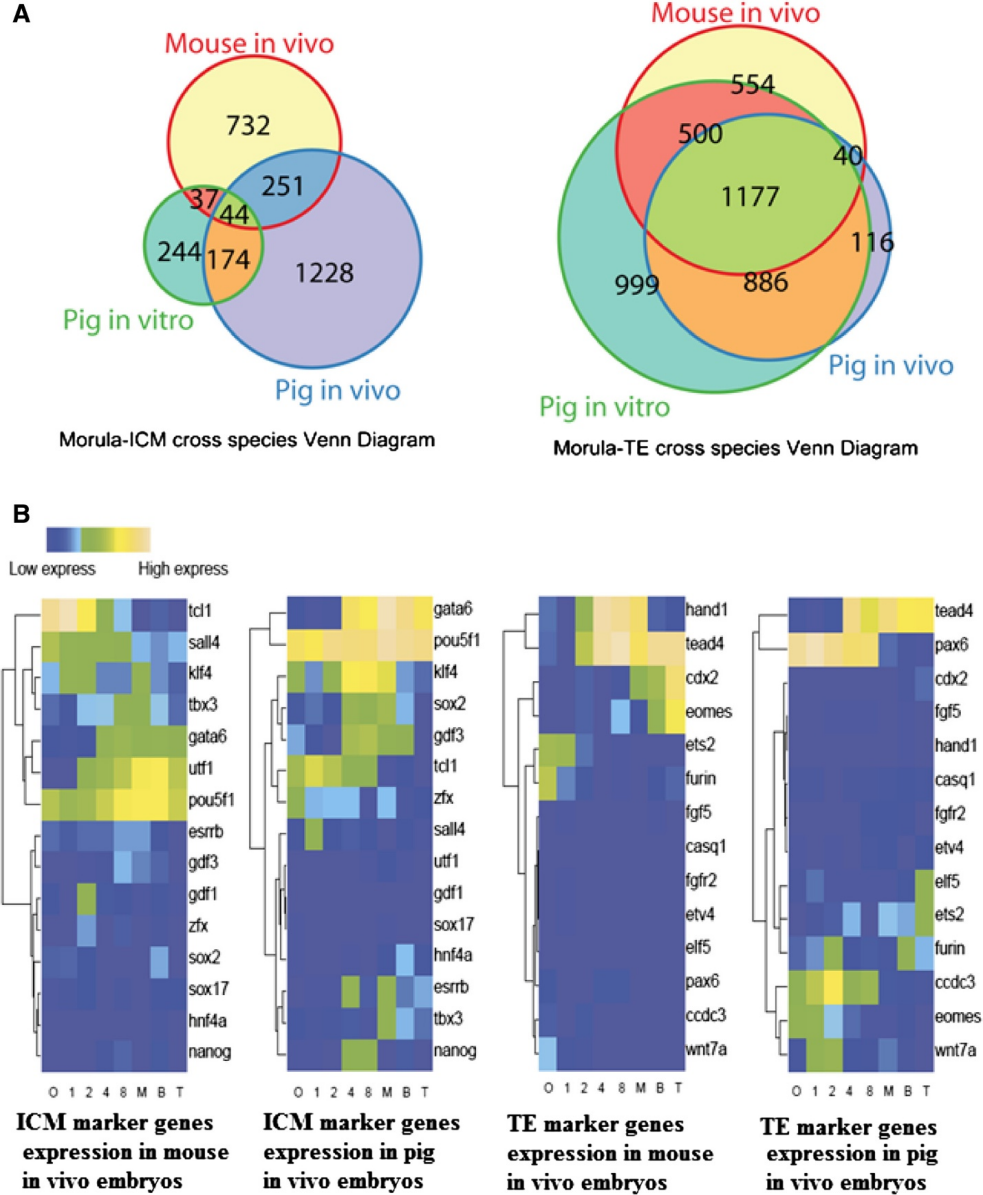
**Gene expression profiling for lineage segregation during PED. (A)** Venn diagram of co-expression genes in the morula and ICM (left), and in the morula and TE (right). Co-expression relationship for one gene is on both lists: 1) morula cells gene expression list (RPKM >3). 2) ICM cells specific gene expression list or TE cells gene expression list (RPKM >3). **(B)** Heat map of ICM and TE marker gene clustering (highly expressed genes are shown in yellow, and minimally expressed genes are shown in blue).

To understand the mechanisms underlying lineage segregation in pig PED, we analyzed co-expressed genes in the morula and ICM and genes shared by the morula and TE in mouse and pig (Figure 4A). Both gene groups may be relevant to ICM development and TE formation independently because both ICM and TE differentiate from the morula.

Among the co-expressed genes in the morula and ICM, we observed that 295 genes (27.7% in ms; 17.3% in Pnm) were shared between Pnm and Ms, including Oct4, Tbx3, Gata6 and Smad4. These genes may be involved in regulating second lineage segregation (Figure 4A left; Additional file 6: Table S5). Using GO analysis of the shared genes co-expressed by the morula and ICM between the mouse and pig, we found that most gene expression is associated with embryonic biology, including *in utero* embryonic and endoderm development. Mouse and pig embryos also differed in co-expressed genes related to fatty acid metabolic processes, lipid metabolic processes, and biological aspects of the cytoplasm, nucleus, mitochondria, and protein binding (Additional file 6: Table S5).

A total of 1,217 genes were commonly expressed in the morula and TE cells in Ms and in Pnm (53.6% in mice and 54.8% in pigs) (Figure 4A right). Genes related to lineage commitment, such as Tcf3, Tcfap2c, Cnot7, Grb2 and Smad2, were found in both pigs and mice. Mouse morula and TE co-expressed genes included Cdx2, Fgfr1op, Tcf15, Trim11, Trim 27, Eed, Gata3, Fgfr1, Cnot8, Grb7, and Tcf25. Pnm morula and TE co-expressed genes included Trim15, Trim37, Trim35, Gata2, Fgf7 and Grb10 but not Cdx2. (Additional file 6: Table S5). These data indicate that TE lineage differentiation is regulated by different signaling pathways or by different levels of gene expression in pig and mouse embryos. Comparative analysis of the expression of specific markers of ICM and TE in the different stages of embryos was performed (Figure 4B and Additional file 2: Figure S6). A significant difference in the heat map patterns for these marker genes suggests that regulation of ICM development among pigs, mice and the human were more conserved than those of TE development.

### Expression of enzymes relevant to fatty acid metabolism in pig early embryonic development

**Figure 5:**
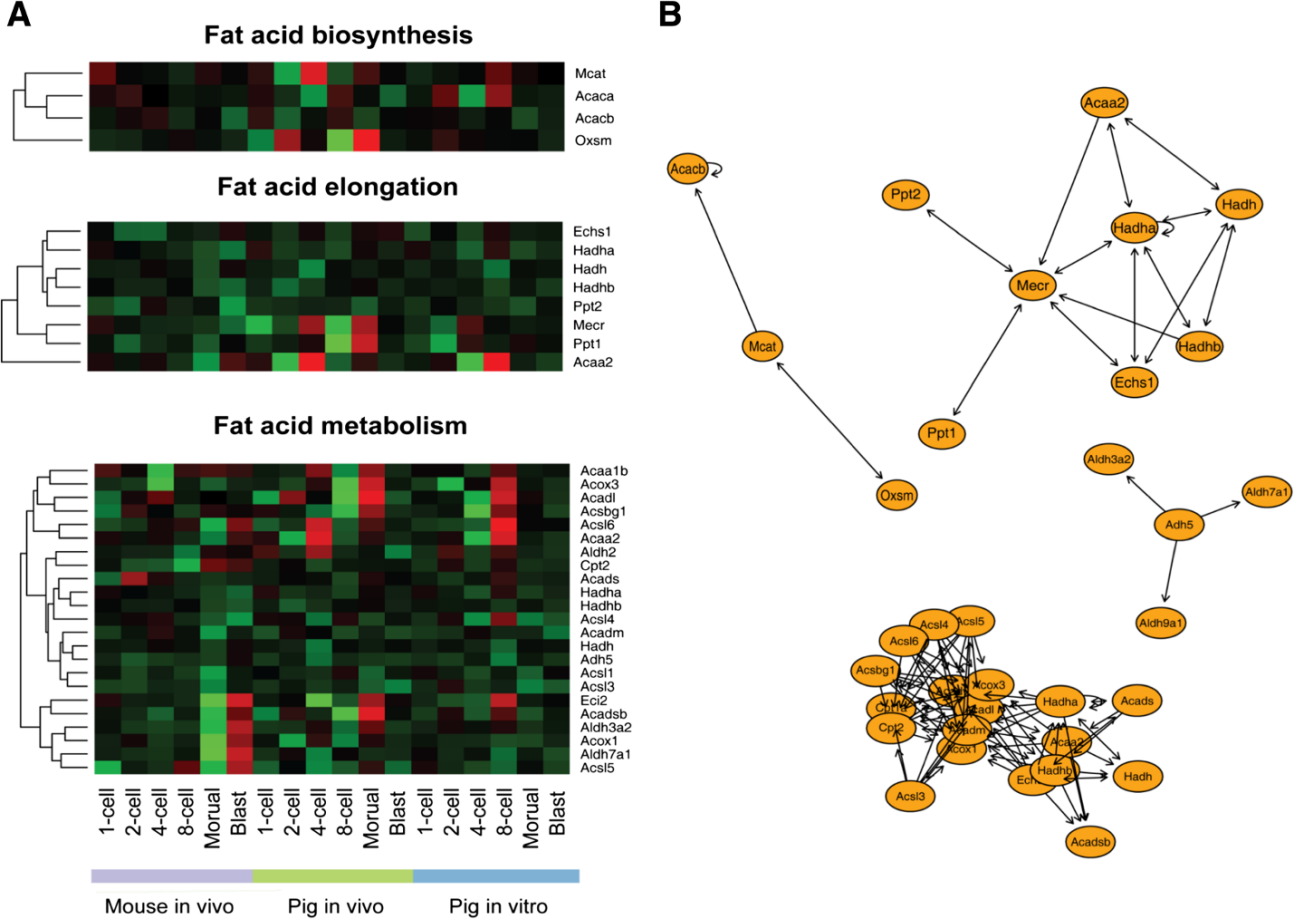
**Fatty acid metabolism of pig PED. (A)** Fatty acid pathways in pre-implantation embryos. Three fatty acid-related sub-pathways from the KEGG database are shown in hierarchical clusters, and genes with one-to-one orthologous relationships between pigs and mice were selected. **(B)** Network of fatty acid related genes drawn using the KEGG graph package [33]. This network depicts the relative interaction relationships for those enzymes. Directly linked enzymes were more closely related than indirectly linked enzymes.

Comparing gene expression in mouse and pig embryos and their relevant pathways during PED, we observed an enrichment in the pathways relevant to fatty acid metabolism during ZGA and lineage segregation in pig embryos. We then evaluated the pathways of fatty acid biosynthesis, fatty acid elongation, and fatty acid metabolism during PED. Genes for enzymes that regulate fatty acid biosynthesis and elongation were highly expressed at the 4-cell and the morula stage in normal pig embryos, compared with mouse and pig SCNT embryos. In contrast, a slight increase in expression of these enzymes was observed in mouse blastocysts and in pig 8-cell SCNT embryos (Figure 5; Additional file 7: Table S6). Thus, fatty acid biosynthesis and elongation in pre-implantation embryos may be important to pig embryonic and fetal development. In addition, enzymes related to fatty acids are activated during different stages of development and at different levels across species. Under current culture conditions, oocytes for pig SCNT embryos were allowed to mature *in vitro*, and the reconstructed embryos were cultured in fatty-acid-free medium. This may explain the low efficiency of pig cloning. Further studies are warranted to understand the mechanisms by which fatty acid metabolism is regulated during pig PED and in *in vitro* culture of pig pre-implantation embryos, ES cells and iPSCs.

### Nuclear transfer micromanipulation and global gene expression patterns during reconstructed embryo development

The ooplasm contains factors that erase somatic epigenetic imprints, rendering the somatic nucleus totipotent. Gene expression patterns that occur after nuclear transfer may be essential to later development. Identification and comparison of the unique gene expression patterns of normal and cloned pig embryos may help determine the unique requirements of cloned embryos and inform us about specific factors in pig iPSCs reprogramming.

To explore mechanisms underlying reprogramming of SCNT, we analyzed serial gene expression of normal and cloned embryos at different development stages. The correlation coefficient between normal ICMTE and SCNT embryonic ICMTE was found to be 0.93 (Additional file 3: Table S2). We observed fewer putative ICM-specific genes in the cloned embryos than in the normal embryos (Figure 3B). Perhaps cloned embryos contain fewer ICM cells than normal embryos [34, 35]. GO analysis of genes expressed in cloned embryonic putative ICM showed that they participated in pathways involving protein binding and regulation in the cytoplasm, mitochondria and nucleus, presumably because of interactions between the ooplasm and the transferred somatic nucleus. However, genes expressed in normal embryos were related to signaling pathways involving the cytoplasm, nucleus, mitochondria, ATP binding, nucleotide binding and fatty acid metabolic processes (Additional file 2: Figure S5B; Additional file 5: Table S4). Fatty acid metabolism appears to be unique to pig early embryonic development, and its function is unclear. In addition, pig normal and SCNT embryos differed in signaling pathways relevant to development including FGF, MAPK, Jak-Stat, and Wnt pathways (Additional file 2: Figure S9).

In mouse SCNT, functional defects in the trophoblast cell lineage are the main cause of low cloning success rates [36]. Pnm and Pnt comparative analysis of the transcriptomes of embryos at different stages revealed that the correlation of TE from blastocysts is lower than that of ICMTE (Figure 1C). GO analysis revealed differences in biological processes in the nucleus, cytoplasm, and cell organs between normal and cloned embryonic TE (Additional file 8: Table S7). This suggests that the functional defects in the trophoblastic cell lineage might also contribute to low efficiency in pig cloning.

### Putative gene regulation networks during pig PED

From our data, we assumed that most of the two lineage segregations during PED were conserved across the three-layer networks of mouse and pig embryos collected *in vivo*. Marker genes (§ Experimental procedures) for the three layers were selected based on previous findings [18, 37]. Co-expression networks of those marker genes were constructed based on the Pearson correlation coefficient (Additional file 2: Figures S10A and B), and singleton sub-networks (common targeted genes from two distinct marker genes that are less than 5% we defined as a singleton network) were removed from the final network.

Network evaluation revealed that pig and mouse pre-implantation embryos share some hub genes (about 50%) with regulatory networks for lineage segregations. In sub-network analysis, ICM-lineage segregation and primitive endoderm differentiation markers such as Esrrb, Gata6, Pou5f1, Tcl1, Sall4 and Tbx3, and TE commitment makers such as Hand1, Ccdc3, Ets2, Wnt7a and Eomes, were found to be common to pigs and mice (Additional file 2: Figures S10A and B). KEGG pathway analysis of all of the networks involved with these genes revealed that the regulation process in pig embryos is mostly associated metabolic pathways whereas that in mouse embryos, is mostly associated with signal transduction (Additional file 2: Figure S10C and Additional file 9: Table S8). These results also suggest that metabolic pathways, including fatty acid metabolism, are important in lineage segregation during pig PED, although details of the underlying mechanism remain to be further explored.

## Discussion

We developed a platform for mRNA sequencing of porcine pre-implantation embryos and a method for evaluating putative ICM gene expression by comparing the ICMTE and TE transcriptome. Genome-wide transcriptome analysis revealed unique molecular gene regulation networks which regulate ZGA, lineage segregation and embryonic metabolism during pig PED.

Some highly expressed genes in pig putative ICM, most of which are related to ES cell pluripotency, differed from those highly expressed in mouse ICM [18]. Even with shared pathways that occur at the same stage of PED, the specific members of special signaling pathways that are highly expressed were different between mice and pigs. This indicates that pig PED is regulated by a species-specific network of genetic factors. Identification of the activated genes in ES cell-specific pathways such as the TGF-beta, MAPK, Jak-Stat and Wnt pathways support this conclusion. SCNT is a reliable platform for studying somatic cell reprogramming. Differences between the Pnm and Pnt embryos, especially between their ICMs, provide insights into the mechanisms involved in somatic reprogramming, including those involved in the generation of iPSCs. For example, we found that fatty acid metabolic processes to be significantly more active in Pnm than in mouse or SCNT pig pre-implantation embryos. These results indicate that fatty acid metabolism may be important for pig PED and lineage segregation events. It may also be important for the maintenance of pluripotency, which may be a useful nutritional element during derivation of true pig ES cell lines.

The molecular regulators of early lineage segregation in mouse embryos are well known but those in pig embryos are not. Transcriptome analysis of genes co-expressed by the morula and ICM, and co-expressed by the morula and TE in the pig and comparison with genes common to those in mice revealed that some markers of early embryonic segregation, such as Cdx2 and Nanog, may not be restricted to the ICM or the TE in the pig blastocyst. This indicates that either a different mechanism regulates the differentiation of the pig pre-implantation embryonic ectoderm lineage or these events occur at a later stage in pig PED. Analysis of genes co-expressed by the morula and ICM together with morula and TE revealed that regulation of ICM differentiation is more conserved. However, the regulatory mechanism of TE differentiation may be subtly different in mouse and pig embryos because Pnm ICM expresses Eed and Gata3, which are restricted to the TE in mice. It is unclear whether Oct4 expression may limit Cdx2 expression in the pig TE as it does in mouse embryos. It is also unclear if Oct4 expression in pig TE causes a decline in the control of TE differentiation or if there are other factors that are expressed in the Pnm TE that regulate TE differentiation.

The events that occur shortly after fertilization in normal embryos include segregation of maternal chromosomes, breakdown of the sperm’s nuclear envelope, repackaging of the sperm’s chromatin, and the formation of two pronuclei. In cloned embryos, the sub-cellular changes include the breakdown of the somatic cell nuclear envelope, the condensation of the somatic cell chromosomes, and the formation of pseudo-pronucleus [30]. The different processes that occur during this transient period may cause gene expression patterns to differ between normal and cloned embryos, which may then affect the regulation of later development. Aberrant global gene expression and delay of the ZGA in SCNT pre-implantation embryos may be the reasons for the incomplete reprogramming observed in SCNT embryos.

Evidence from previous studies indicates that individual fatty acids may affect oocyte maturation and embryonic development [38, 39]. Endogenous lipids are more abundant in pig oocytes and pre-implantation embryos than in mouse, cattle and sheep. This can cause significant damage during cryopreservation [40]. Lipids have important functions in membrane receptor biology, signal transduction, and growth regulation [41–46]. However, little is known about the fatty acid metabolism network and its regulation in pre-implantation embryos. Information concerning lipid uptake and utilization by oocytes and embryos may therefore be crucial to improving cryopreservation practices, *in vitro* culture systems for the derivation of pig ESCs, and the combination of the factors used for reprogramming into pig iPSCs.

## Conclusion

In summary, our study represents a significant step in the characterization of pig pre-implantation embryos and provides insight into the dynamic molecular regulation of pig PED. We provide genome-level evidence and gene expression patterns for events such as ZGA and lineage segregation during PED. GO and KEGG analyses of each trancriptome suggests that pathways which regulate epiblast and primitive endoderm lineage commitment may be more conserved than those that regulate ectoderm segregation. Our data also provide a resource for pig pluripotent stem cell engineering and for understanding porcine development.

## Methods

### Embryo collection and RNA isolation

Young adult female C57 mice (Vital River Laboratories, Beijing, China) and young adult female Nong Da Xiang pigs (China Agricultural University pig farm, Zhuo Zhou, China) were kept in a 12 h light/dark cycle and given water and food *ad libitum*. All mice and pigs were handled and studies were carried out according to the guidelines of The State Key Laboratory Animal Care and Use Committee.

Oocytes were collected from the oviduct of C57 mice 14 h after injection with human chorionic gonadotropin (hCG). Then pre-implantation embryos were collected at various points after hCG injection and mating as depicted in Table 1. Pure TE was separated physically using an ultra-sharp splitting blade (Bioniche, Animal Health US, Inc) under a stereomicroscope (Additional file 2: Figure S1).

**Table 1:** **Collection schedule of the pre-implantation embryos at different developmental stages**

	***Oocyte***	***1-cell***	***2-cell***	***4-cell***	***8-cell***	***Morula***	***ICM***	***TE***
Mouse embryos*	14 h	24–26 h	36 h	48 h	60 h	72 h	96 h	96 h
Pig *in vivo* embryos**	24 h after estrus	24 h	40–45 h	65–72 h	84–90 h	108–115 h	156–160 h	156–160 h
Pig SCNT embryos***	*In vitro* matured	24 h	40 h	65–72 h	84–90 h	108–115 h	156–160 h	156–160 h

*collected N hours after hCG injection; **collected N hours after natural mating; ***collected N hours after activation.

Normal pig embryos were washed from the oviduct or uterus using PBS with 5% FBS at the indicated time points (Table 1) after estrus and natural mating. Donors of normal embryos were mini pigs (Nong Da Xiang, a local strain), and the same strain was used to harvest fibroblasts that were used as donor cells for SCNT. SCNT embryos were collected after activation of reconstructed embryos at time points given in Additional file 1: Table S1.

Next, 4–10 embryos at the same stage were pooled together for each sample and transferred into extraction buffer from the PicoPure RNA Isolation Kit (Arcturus, KIT0204, Life Technologies, US) at 42°C for 30 min. Samples were either stored at −80°C for up to one month or used immediately for analysis. RNA from each sample was extracted according to the manufacturer’s instructions and eluted into 10-μL elution buffer.

### mRNA sequencing

We performed mRNA sequencing using the Applied Biosystems SOLiD 4 System as follows: RNAs isolated from pre-implantation embryos were used for double-stranded cDNA synthesis and PCR amplification. The procedure for fragment library preparations was performed according to the Library Preparation Protocol for whole transcriptome analysis of a single cell (http://www.appliedbiosystems.com) and the Applied Biosystems SOLiD 4 System Library Preparation Guide (http://www.appliedbiosystems.com). mRNA sequencing data are available from the Lab Archive (http://www.ncbi.nlm.nih.gov/sra) under accession number SRA076823.

### Transcriptome analysis

Transcriptome analysis tools included in the BioScope software package (Applied Biosystems) were used to map the corresponding sequenced reads against the mouse mm9 genome and the pig susScr2 genome. Mouse (NCBIM37.65) and pig (Sscrofa9.65) gene and transcript annotation files were downloaded from the Ensembl database (http://asia.ensembl.org/info/data/ftp/index.html).

Prior to differential gene expression analysis, read count tables were generated from binary sequence alignment/map (BAM) files using HTseq software (http://pypi.python.org/pypi/HTSeq). The value for reads per kilobase of coding sequence per million mapped reads (RPKM) was calculated to estimate gene expression under each set of conditions [47]. For each sequenced library, the read counts were adjusted using the edgeR software package through a one-scaling normalized factor [48, 49]. The DEGseq software package was used to calculate differences in gene expression between the two assigned groups. A gene was considered significant if the Benjamini and Hochberg corrected *P*-value was less than 0.05 and the fold-change was greater than 2 [50]. Differentially expressed isoforms were estimated using Cufflinks v1.30 while treating early pre-implantation embryo samples as a time-series input.

We also downloaded human pre-implantation embryonic datasets from GSE36552, deposited in GEO databases (http://www.ncbi.nlm.nih.gov/geo/) [21]. For each developmental embryonic stage, pooled read counts were used for recalculating RPKM values. EPI, PE and TE single cells datasets in blastocysts were analyzed as described in this paper. All genes with RPKM > 0.1 were defined as expressed in individual cells at same stage. Based on our results, we then compared pig and mouse *in vivo* datasets with human PED datasets.

Gene ontology (GO) was implemented using the GOseq software package, in which gene length bias was adjusted [51]. The BioMart system was used to abstract orthologous relationships between pig and mouse (one-to-one) gene pairs and to convert mapping information regarding gene identities between mouse Ensembl and reference sequences [52, 53].

ICM segregation markers included Pou5f1, Sox2, Klf2, Nanog, Rex1, Utf1, Zfx, Esrrb, Tbx3, Tcl1 and Klf4. Primitive endoderm differentiation markers included Gata6, Gata4, Gdf1, Gdf3, Hnf4a (Nr2a1), Mixl1, Sall4, Sox7 and Sox17. TE commitment markers included Cdx2, Eomes, Hand1, Fgfr2, Ets2, Tcfap2, Elf5, Etv4, Furin, Ccdc3, Pace4, Casq1, Wnt7a, Fgf5, Pax6, and Tead4 [18, 37]. Only genes with orthologous relationships between pigs and mice were kept for analysis (Klf2, Rex1, Gata4, Mixl1, Sox7, and Pace4 were removed). Three types of markers were chosen for co-expression sub-network construction analysis, which were displayed graphically using Cytoscape 2.8 [54]. The absolute value of the correlation between expression profiles includes all points in time during embryonic development. The correlation between markers and predicted targets must be larger than 0.9.

### Whole-mount immunofluorescence

Embryos were fixed in 4% paraformaldehyde in washing solution (PBS with 0.1% Tween 20 and 0.01% Triton X-100) for 30 min at room temperature. Then embryos were permeabilized in 1% Triton X-100 in PBS for 4 h at 4°C, blocked with 1% BSA in washing solution (blocking solution), and incubated with the following antibodies: Oct4 (Santacruz Sc-8628), Cdx2 (Biogenex MU392A-UC), Nanog (Abnova PAB6837), Sox2 (Santacruz Sc-17320) and Gata6 (Abcam Ab22600) in blocking solution for 1 h at room temperature. After incubation with secondary antibody for 1 h at room temperature, embryos were counterstained with 10 μg/mL Hoechst 3342 (Sigma B2261) in washing solution for 10 min at room temperature.

## Electronic supplementary material

Additional file 1: Table S1: Primary records of samples. (DOC 60 KB)

Additional file 2: **Supplement figures.** **Figure S1.** Splitting of pig blastocyst with an ultra-sharp splitting blade. **Figure S2.** (A) Unsupervised hierarchical clustering of the expression profiles [21]. **Figure S3.** Dnmt3b and Dnmt1 expression. **Figure S4.** Heat map of protein binding-associated transcripts under different conditions. **Figure S5.** Analysis of the ICM-specific genes in mouse and pig. **Figure S6.** Heat map of ICM and TE marker gene clustering in human pre-implantation embryos. **Figure S7.** Heat map clusters of ICM-specific genes in TGF-beta, MAPK, Jak-Stat and Wnt signaling pathways expressed during normal mouse PED and normal pig PED. **Figure S8.** Immunostaining of the pluripotent markers in pig and mouse pre-implantation embryos. **Figure S9.** Heat map clusters of ICM-specific genes in TGF-beta, MAPK, Jak-Stat and Wnt signaling pathways expressed during normal pig *in vivo* and SCNT pig PED. **Figure S10.** Putative regulatory networks for lineage segregation during PED. (PDF 1 MB)

Additional file 3: Table S2: Sample correlation. (XLSX 14 KB)

Additional file 4: Table S3: Identification of maternal deposition and zygotic gene activation. (XLS 836 KB)

Additional file 5: Table S4: Gene expression profiling in putative ICM. (XLS 58 KB)

Additional file 6: Table S5: Gene profiling of regulation of lineage commitment during PED. (XLS 836 KB)

Additional file 7: Table S6: Unique fatty acid metabolism during pig PED. (XLS 582 KB)

Additional file 8: Table S7: TE expression and GO analysis. (XLSX 114 KB)

Additional file 9: Table S8: Putative regulation networks during PED. (XLS 582 KB)
