# Supplementary material for: Specific gene-regulation networks during the pre-implantation development of the pig embryo as revealed by deep sequencing
Source: BMC Genomics. 2014 Jan 3;15(1):4. doi: 10.1186/1471-2164-15-4 (PMC3925986; doi:10.1186/1471-2164-15-4)
Supplement: Supplementary file 1 — Additional file 1: Table S1: Primary records of samples. (DOC 60 KB) [file 12864_2013_6998_MOESM1_ESM.doc]

Table S1 Primary Record of Samples

| **Species** | **First Samples** | **Number** | **Second Samples** | **Number** |
| --- | --- | --- | --- | --- |
| Mouse | oocyte | 30 |  |  |
| Mouse |  |  | 1 cell | 15 |
| Mouse | 2 cell | 10 |  |  |
| Mouse | 4 cell | 6 |  |  |
| Mouse | 8 cell | 5 |  |  |
| Mouse | morula d3 | 6 |  |  |
| Mouse | ICM d4 | 6 |  |  |
| Mouse | TE d4 | 4 |  |  |
| Pig Normal | In vivo Oocyte | 12 | In vivo Oocyte | 15 |
| Pig Normal |  |  | In vivo 1 cell | 8 and 9 |
| Pig Normal | In vivo 2 cell | 8 | In vivo 2 cell | 5 |
| Pig Normal | In vivo 4cell | 5 | In vivo 4cell | 6 |
| Pig Normal | In vivo 8cell | 6 | In vivo 8cell | 4 |
| Pig Normal | In vivo morula | 4 | In vivo morula | 6 |
| Pig Normal | In vivo ICM | 4 | In vivo ICM | 8 |
| Pig Normal | In vivo TE | 4 | In vivo TE | 4 |
| Pig NT |  |  | NT Oocyte | 30 |
| Pig NT |  |  | NT 1 cell | 30 |
| Pig NT | NT 2 cell | 20 |  |  |
| Pig NT | NT 4 cell | 16 |  |  |
| Pig NT | NT 8 cell | 10 |  |  |
| Pig NT | NT morula | 12 |  |  |
| Pig NT | NT ICM | 15 |  |  |
| Pig NT | NT TE | 10 |  |  |
| Pig iPS cells | iPS-21 P8 |  |  |  |
| Pig iPS cells | iPS-28 P13 |  |  |  |
| Pig iPS cells | iPS G1 P8 |  |  |  |

NT: Nuclear transfered
