# Supplementary material for: Specific gene-regulation networks during the pre-implantation development of the pig embryo as revealed by deep sequencing
Source: BMC Genomics. 2014 Jan 3;15(1):4. doi: 10.1186/1471-2164-15-4 (PMC3925986; doi:10.1186/1471-2164-15-4)
Supplement: Supplementary file 2 — Additional file 2: Supplement figures. Figure S1. Splitting of pig blastocyst with an ultra-sharp splitting blade. Figure S2. (A) Unsupervised hierarchical clustering of the expression profiles [21]. Figure S3. Dnmt3b and Dnmt1 expression. Figure S4. Heat map of protein binding-associated transcripts under different conditions. Figure S5. Analysis of the ICM-specific genes in mouse and pig. Figure S6. Heat map of ICM and TE marker gene clustering in human pre-implantation embryos. Figure S7. Heat map clusters of ICM-specific genes in TGF-beta, MAPK, Jak-Stat and Wnt signaling pathways expressed during normal mouse PED and normal pig PED. Figure S8. Immunostaining of the pluripotent markers in pig and mouse pre-implantation embryos. Figure S9. Heat map clusters of ICM-specific genes in TGF-beta, MAPK, Jak-Stat and Wnt signaling pathways expressed during normal pig in vivo and SCNT pig PED. Figure S10. Putative regulatory networks for lineage segregation during PED. (PDF 1 MB) [file 12864_2013_6998_MOESM2_ESM.pdf]

Additional file 2 Supplement figures

Figure S1. Splitting of pig blastocyst with an ultra-sharp splitting blade under a stereomicroscope. The image on the left contains ICM and TE (here called ICMTE), and the image on the right shows pure TE. **Figure S2.** (A) Unsupervised hierarchical clustering of the expression profiles of normal pig embryos *in vivo* and pig SCNT pre-implantation embryos. (B) Unsupervised hierarchical clustering of the gene expression profiles of human pre-implantation embryos based on data from Yan *et al.* [21]. **Figure S3.** Dnmt3b and Dnmt1 expression was measured with RPKM values based on RNA-sequencing results in embryos derived *in vivo* and pre-implantation embryos derived *in vitro*. **Figure S4.** Heat map of protein binding-associated transcripts under different conditions. (A) Corrected ZGA Venn diagram. Mouse zygotic activation was calculated from the 2-cell stage, but it was calculated from the 4-cell and 8-cell stages in normal pig embryos and in pig SCNT PED, respectively. (B) Unsupervised hierarchical heat map of protein binding-associated transcripts. (C) Unsupervised hierarchical heat map of ATP synthesis -coupled proton transport genes. **Figure S5.** Venn diagram of the ICM-specific genes in our datasets and the previously reported list of genes [18] expressed in the ICM (A), Venn diagram of genes expressed in the mouse ICM, ICMTE, and TE (B), and Heat map of ICM-specific gene clustering. Purple indicates that both genes are ICM-specific in the two compared situations. Green indicates that the second gene is ICM-specific. Blue indicates that the first gene is ICM-specific (C). **Figure S6.** Heat map of ICM and TE marker gene clustering in human pre-implantation embryos based on Yan *et al.* [21] data (highly expressed genes are shown in yellow and minimally expressed genes are shown in blue). **Figure S7.** Heat map clusters of ICM-specific genes in TGF-beta, MAPK, Jak-Stat and Wnt signaling pathways expressed during normal mouse PED and normal pig PED. The four pathway candidates were determined from KEGG databases, and only genes that exhibited one-to-one orthologous relationships between pigs and mice were selected. Color intensity was calculated by the fold change of blastocyst RPKM value and TE RPKM value. Red indicates a positive fold change value, and green indicates a negative change in value. **Figure S8.** Immunostaining of the pluripotent markers in pig and mouse pre-implantation embryos. (A) Oct4 and Cdx2 immunostaining in the mouse blastocyst. (B) Cdx2 immunostaining at different stages of porcine PED. (C) Nanog immunostaining at the morula and blastocyst stages of porcine PED. (D) Gata6 immunostaining at the morula and blastocyst stages of porcine PED. (E) Sox2 immunostaining at the blastocyst stage (left). Sox2 expression level was measured using RPKM values and RNA-sequencing results (right). **Figure S9.** Heat map clusters of ICM-specific genes in TGF-beta, MAPK, Jak-Stat and Wnt signaling pathways expressed during normal pig *in vivo* and SCNT pig PED. **Figure S10.** Putative regulatory networks for lineage segregation during PED. ICM lineage segregation and primitive endoderm differentiation markers (diamond shape, red), TE commitment markers (diamond shape, blue) and their predicted targeted genes (round shape) were co-expressed in a sub-network: (A) mouse *in vivo*; (B) pig *in vivo*. (C) KEGG pathway enrichment analysis of each sub-network involved targeted genes ($P < 0.05$).

Fig S1

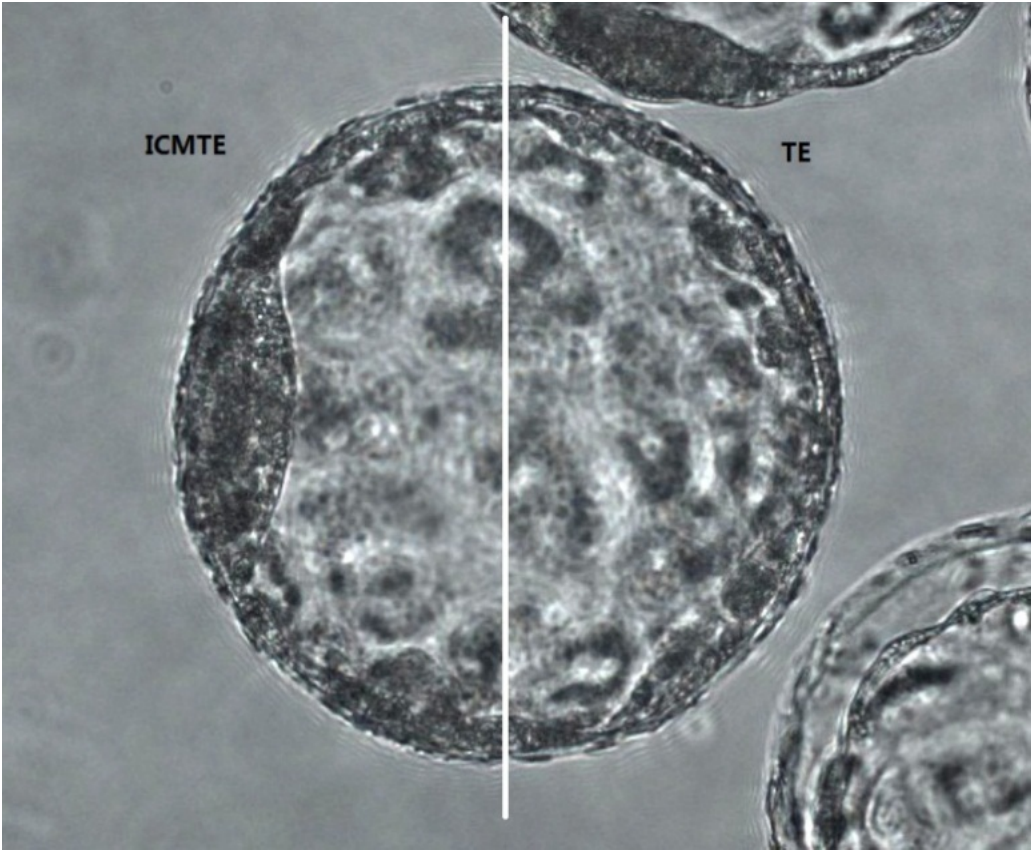

Fig S2

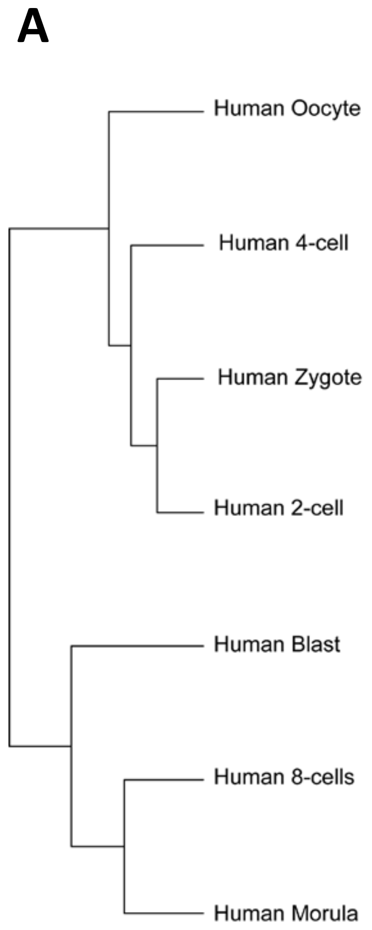

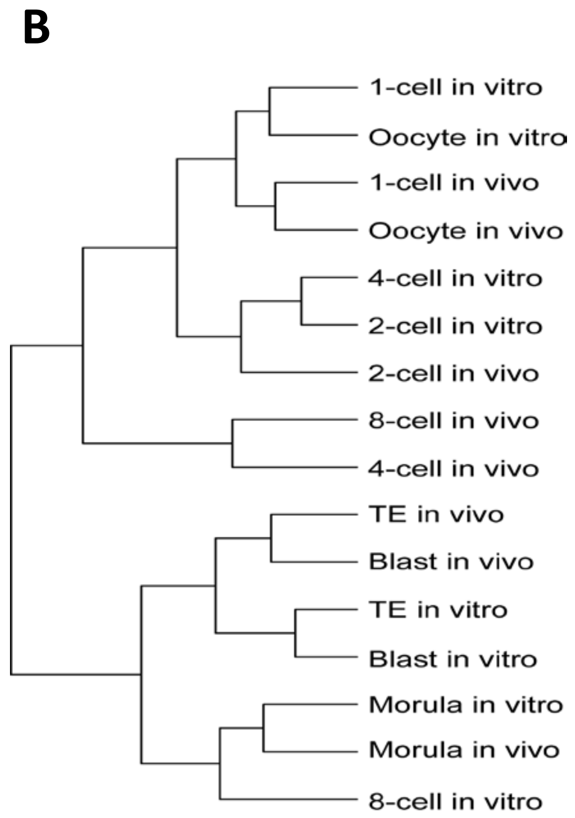

Tang FC, ***nature structural & molecular biology*** , 2013

Fig S 3

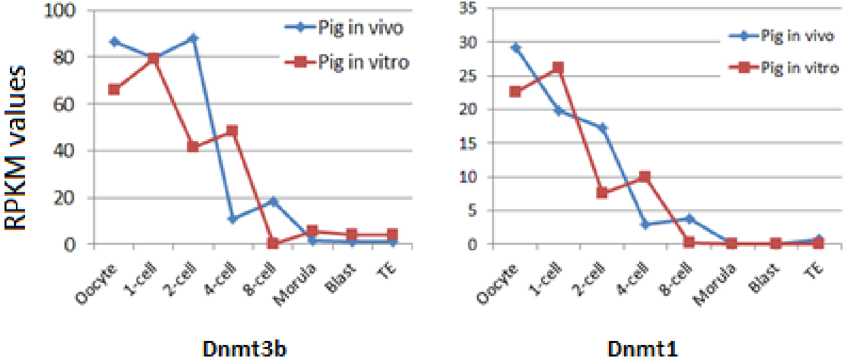

RPKM values
100
80
60
40
20
0
Pig in vivo
Pig in vitro
Oocyte
1-cell
2-cell
4-cell
8-cell
Morula
Blast
TE
Dnmt3b
35
30
25
20
15
10
5
0
Pig in vivo
Pig in vitro
Oocyte
1-cell
2-cell
4-cell
8-cell
Morula
Blast
TE
Dnmt1

Fig S 4

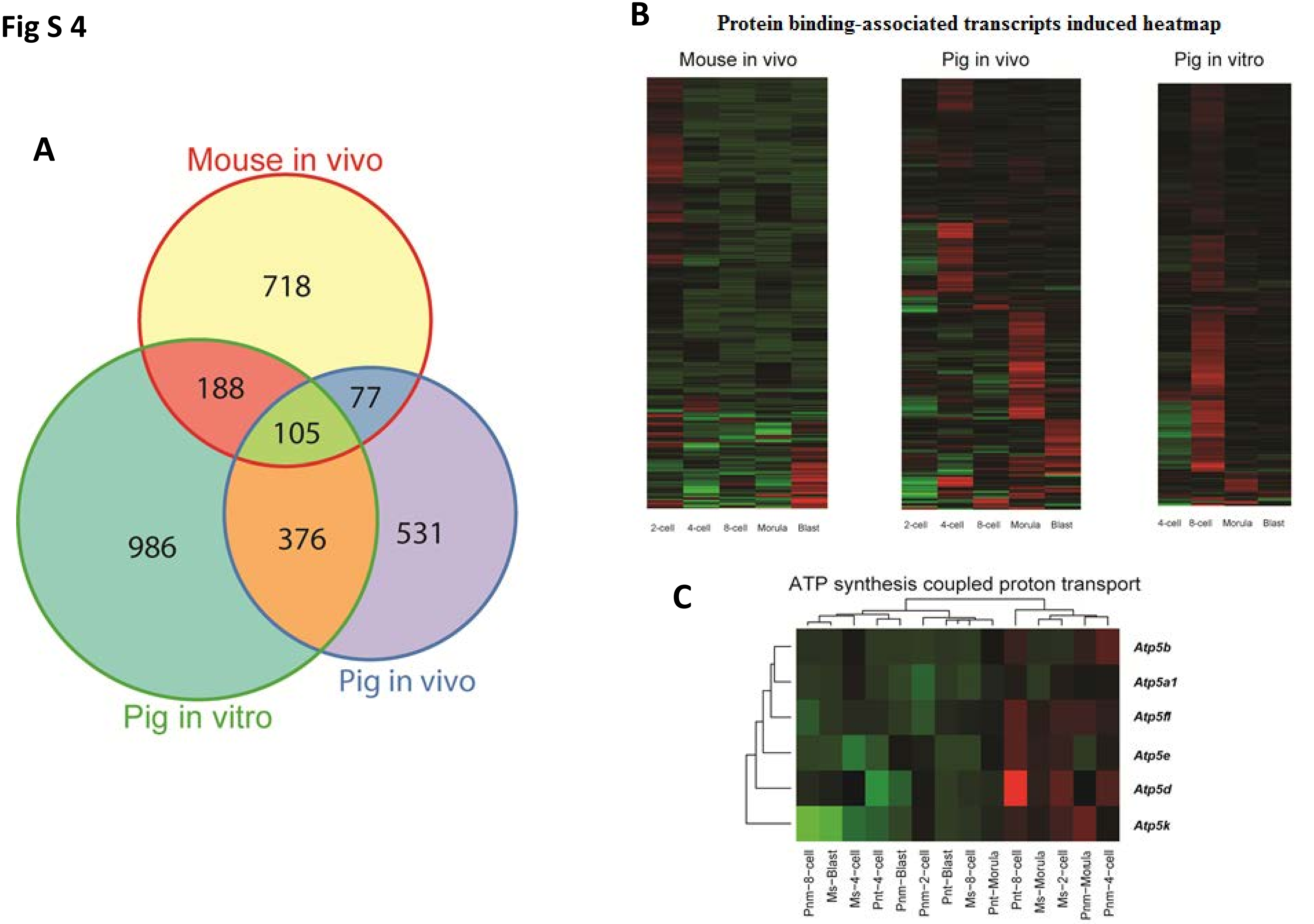
A
Mouse in vivo
718
188
77
105
986
376
531
Pig in vitro
Pig in vivo
B
Protein binding-associated transcripts induced heatmap
Mouse in vivo
Pig in vivo
Pig in vitro
2-cell 4-cell 8-cell Morula Blast
2-cell 4-cell 8-cell Morula Blast
4-cell 8-cell Morula Blast
C
ATP synthesis coupled proton transport
Atp5b
Atp5a1
Atp5fl
Atp5e
Atp5d
Atp5k
Pnm-8-cell
Ms-Blast
Ms-4-cell
Pnt-4-cell
Pnm-Blast
Pnm-2-cell
Pnt-Blast
Ms-8-cell
Pnt-Morula
Pnt-8-cell
Ms-Morula
Ms-2-cell
Pnm-Morula
Pnm-4-cell

Fig S5

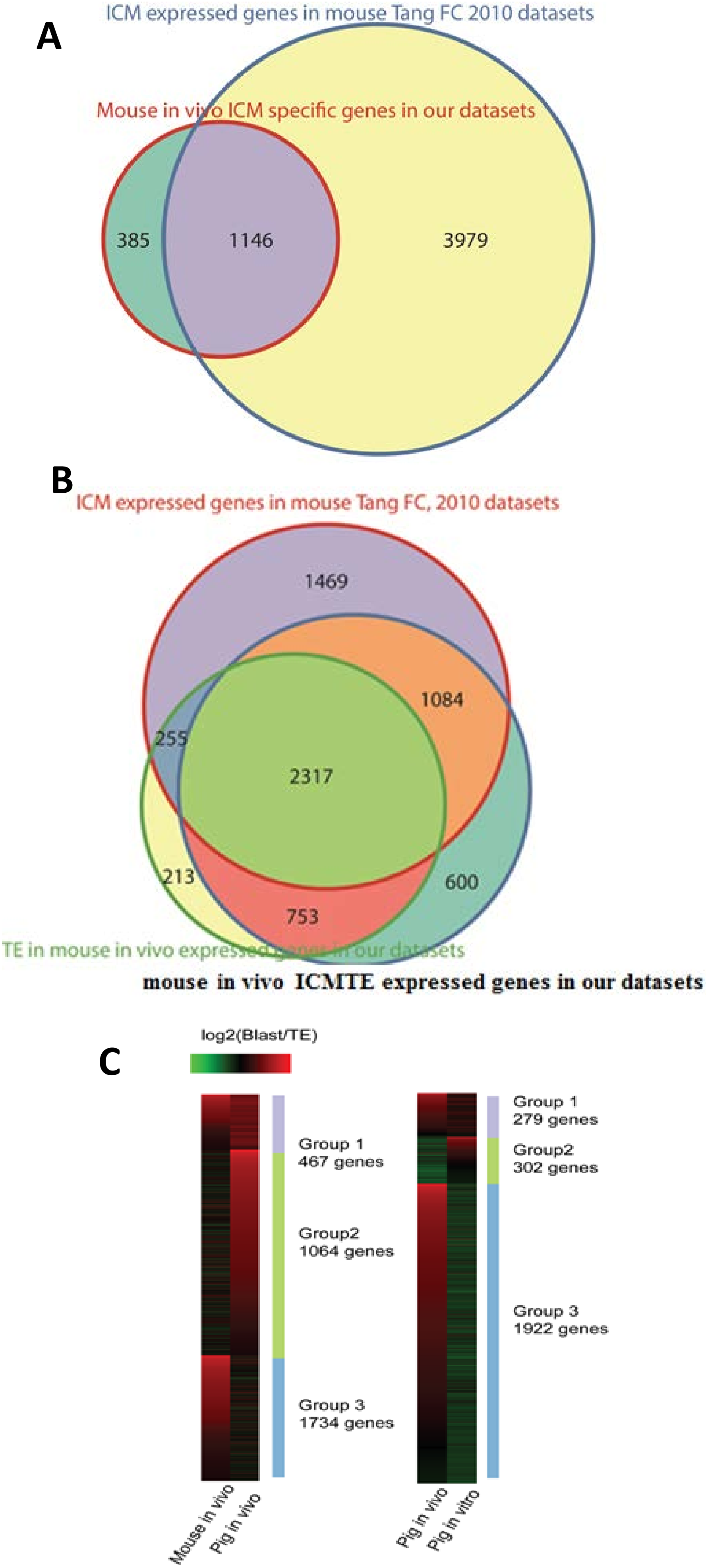
A
ICM expressed genes in mouse Tang FC 2010 datasets
Mouse in vivo ICM specific genes in our datasets
385
1146
3979
B
ICM expressed genes in mouse Tang FC, 2010 datasets
1469
1084
255
2317
213
600
753
TE in mouse in vivo expressed genes in our datasets
mouse in vivo ICMTE expressed genes in our datasets
C
log2(Blast/TE)
Group 1
467 genes
Group2
1064 genes
Group 3
1734 genes
Mouse in vivo
Pig in vivo
Group 1
279 genes
Group2
302 genes
Group 3
1922 genes
Pig in vivo
Pig in vitro

Fig S6

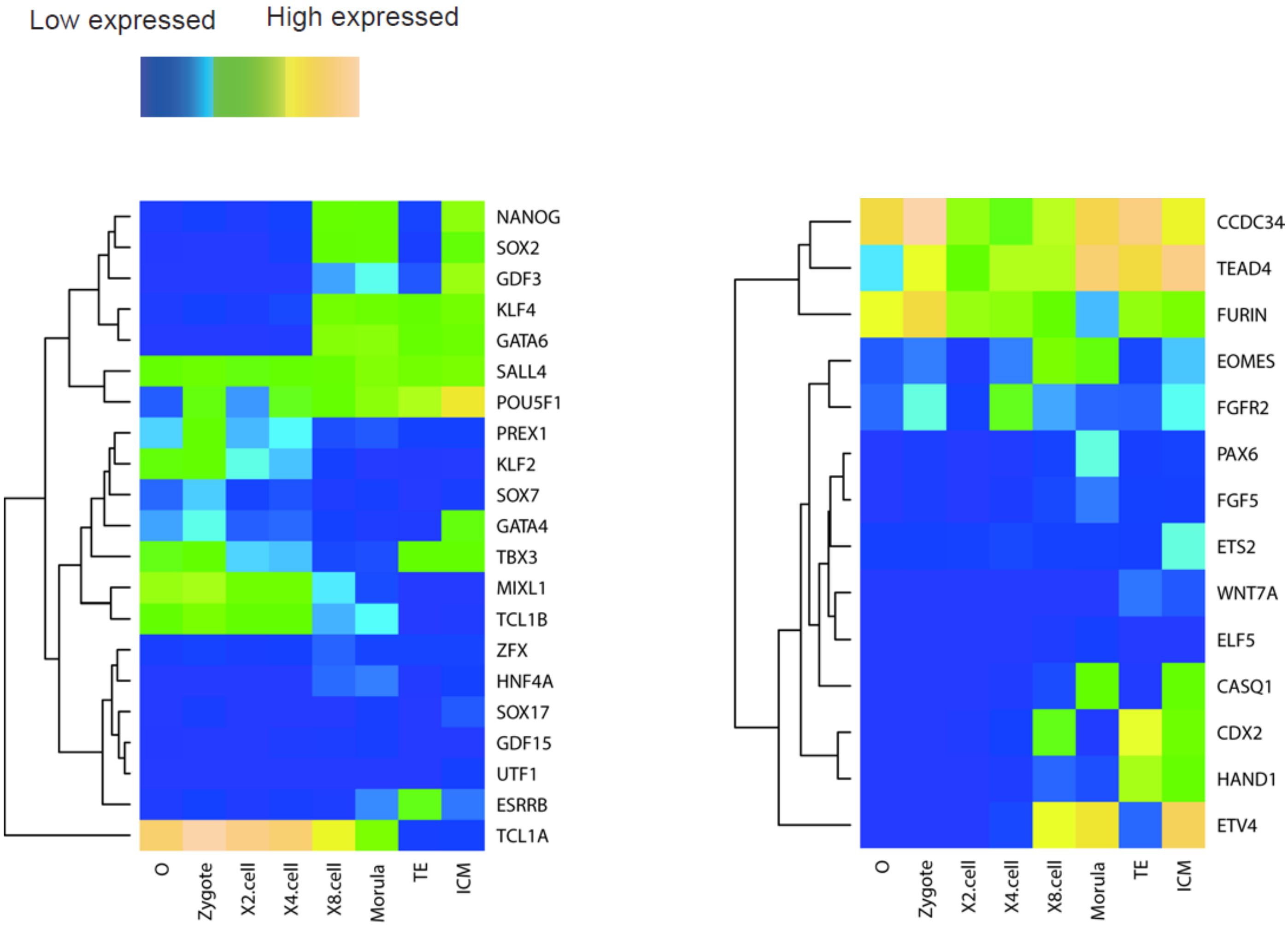

ICM markers expression in human

TE markers expression in human

Fig S 7

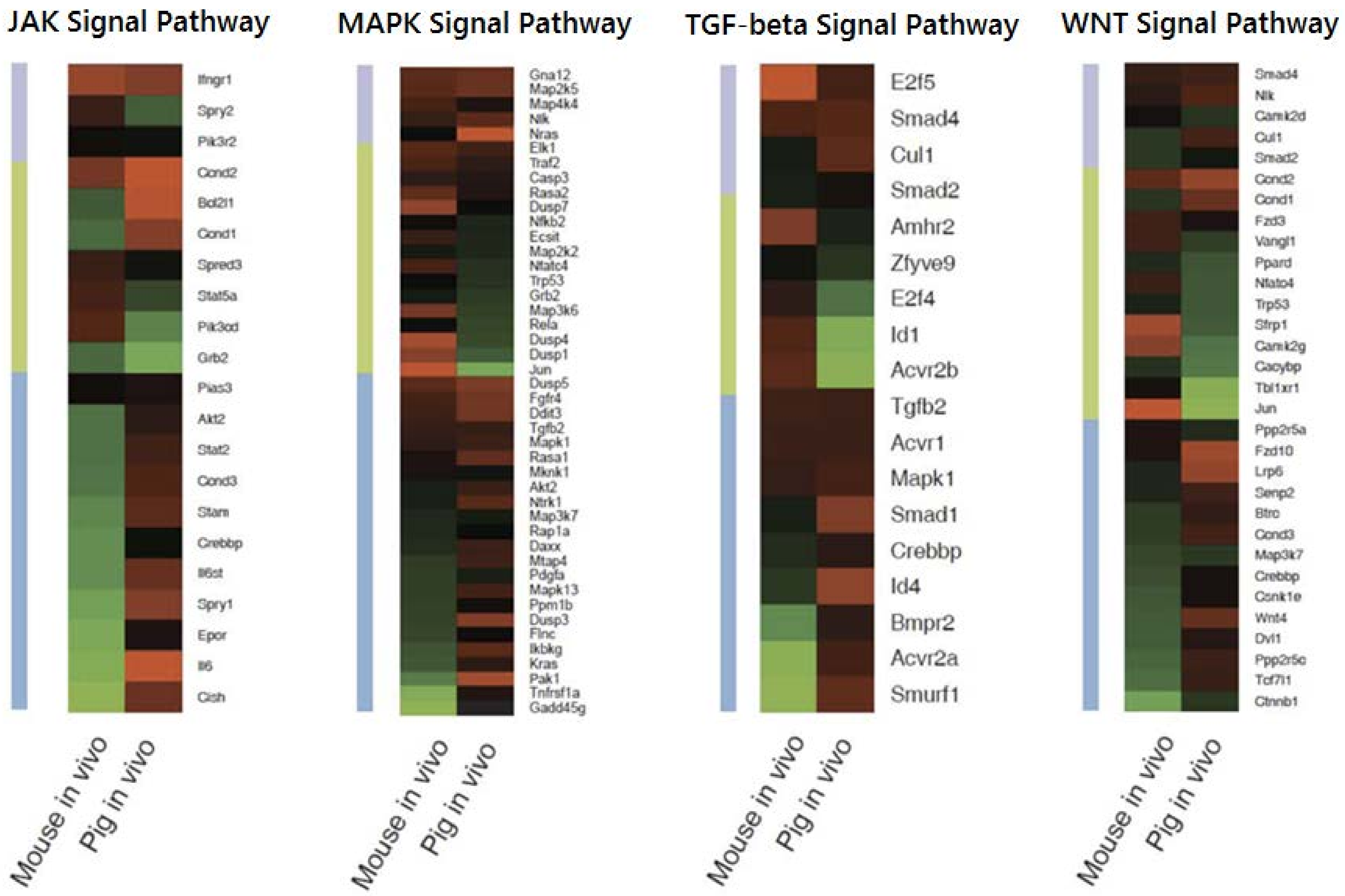
JAK Signal Pathway
Ifngr1
Spry2
Pik3r2
Ccnd2
Bcl2l1
Ccnd1
Spred3
Stat5a
Pik3cd
Grb2
Pias3
Akt2
Stat2
Ccnd3
Stam
Crebbp
Il6st
Spry1
Epor
Il6
Cish
Mouse in vivo
Pig in vivo
MAPK Signal Pathway
Gna12
Map2k5
Map4k4
Nlk
Nras
Elk1
Traf2
Casp3
Rasa2
Dusp7
Nfkb2
Ecsit
Map2k2
Nfatc4
Trp53
Grb2
Map3k6
Rela
Dusp4
Dusp1
Jun
Dusp5
Fgfr4
Ddit3
Tgfb2
Mapk1
Rasa1
Mknk1
Akt2
Ntrk1
Map3k7
Rap1a
Daxx
Mtap4
Pdgfa
Mapk13
Ppm1b
Dusp3
Flnc
Ikbkg
Kras
Pak1
Tnfrsf1a
Gadd45g
Mouse in vivo
Pig in vivo
TGF-beta Signal Pathway
E2f5
Smad4
Cul1
Smad2
Amhr2
Zfyve9
E2f4
Id1
Acvr2b
Tgfb2
Acvr1
Mapk1
Smad1
Crebbp
Id4
Bmpr2
Acvr2a
Smurf1
Mouse in vivo
Pig in vivo
WNT Signal Pathway
Smad4
Nlk
Camk2d
Cul1
Smad2
Ccnd2
Ccnd1
Fzd3
Vangl1
Ppard
Nfatc4
Trp53
Sfrp1
Camk2g
Cacybp
Tbl1xr1
Jun
Ppp2r5a
Fzd10
Lrp6
Senp2
Btrc
Ccnd3
Map3k7
Crebbp
Csnk1e
Wnt4
Dvl1
Ppp2r5c
Tcf7l1
Ctnnb1
Mouse in vivo
Pig in vivo

Fig S 8

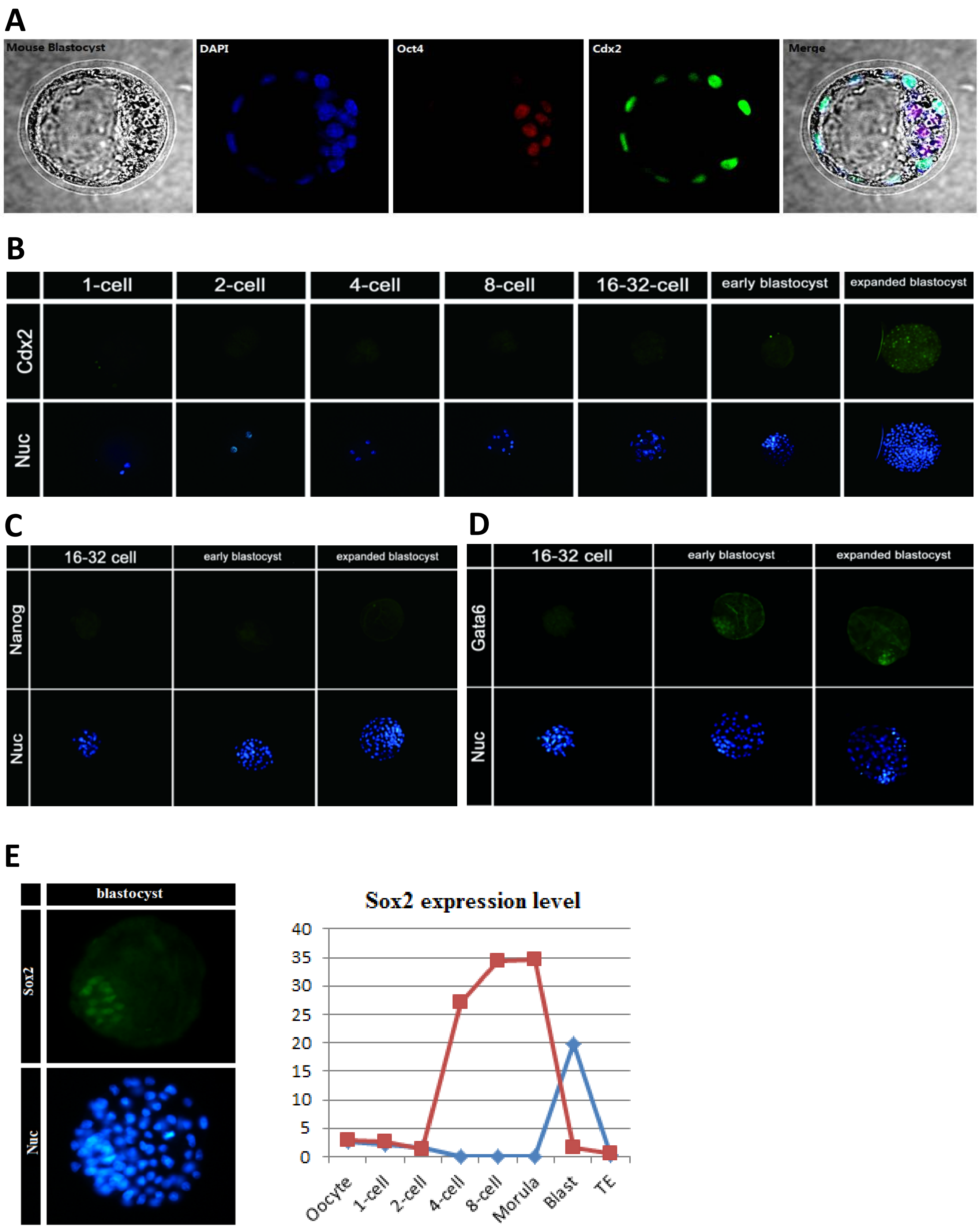
A
Mouse Blastocyst
DAPI
Oct4
Cdx2
Merge
B
1-cell
2-cell
4-cell
8-cell
16-32-cell
early blastocyst
expanded blastocyst
Cdx2
Nuc
C
16-32 cell
early blastocyst
expanded blastocyst
Nanog
Nuc
D
16-32 cell
early blastocyst
expanded blastocyst
Gata6
Nuc
E
blastocyst
Sox2
Nuc
Sox2 expression level
40
35
30
25
20
15
10
5
0
Oocyte
1-cell
2-cell
4-cell
8-cell
Morula
Blast
TE

Fig S 9

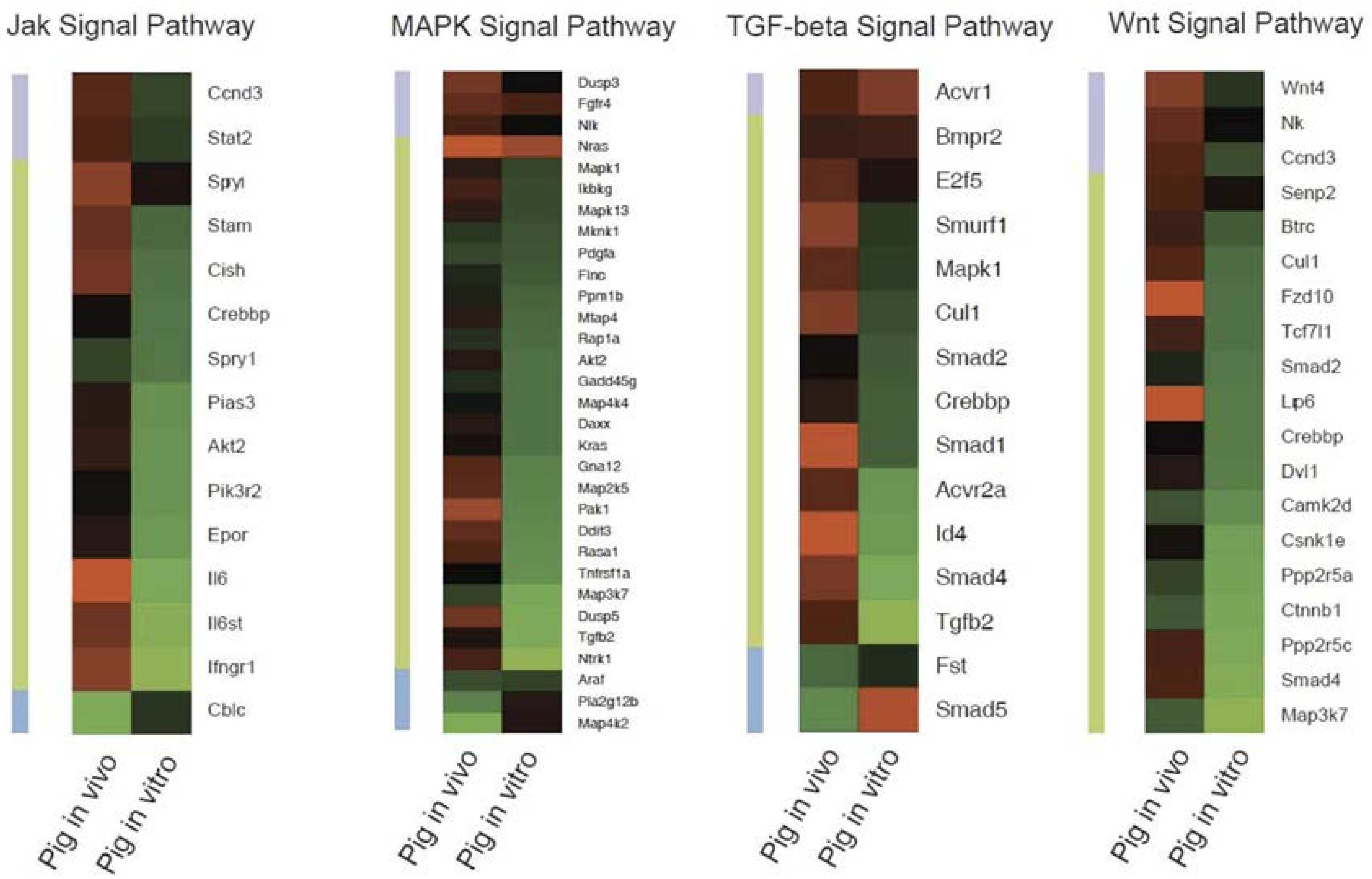
Jak Signal Pathway
Ccnd3
Stat2
Spryl
Stam
Cish
Crebbp
Spry1
Pias3
Akt2
Pik3r2
Epor
Il6
Il6st
Ifngr1
Cblc
Pig in vivo
Pig in vitro
MAPK Signal Pathway
Dusp3
Fgfr4
Nlk
Nras
Mapk1
Ikbkg
Mapk13
Mknk1
Pdgfa
Flnc
Ppm1b
Mtap4
Rap1a
Akt2
Gadd45g
Map4k4
Daxx
Kras
Gna12
Map2k5
Pak1
Ddit3
Rasa1
Tnfrsf1a
Map3k7
Dusp5
Tgfb2
Ntrk1
Araf
Pla2g12b
Map4k2
Pig in vivo
Pig in vitro
TGF-beta Signal Pathway
Acvr1
Bmpr2
E2f5
Smurf1
Mapk1
Cul1
Smad2
Crebbp
Smad1
Acvr2a
Id4
Smad4
Tgfb2
Fst
Smad5
Pig in vivo
Pig in vitro
Wnt Signal Pathway
Wnt4
Nk
Ccnd3
Senp2
Btrc
Cul1
Fzd10
Tcf7l1
Smad2
Lrp6
Crebbp
Dvl1
Camk2d
Csnk1e
Ppp2r5a
Ctnnb1
Ppp2r5c
Smad4
Map3k7
Pig in vivo
Pig in vitro

Fig S10

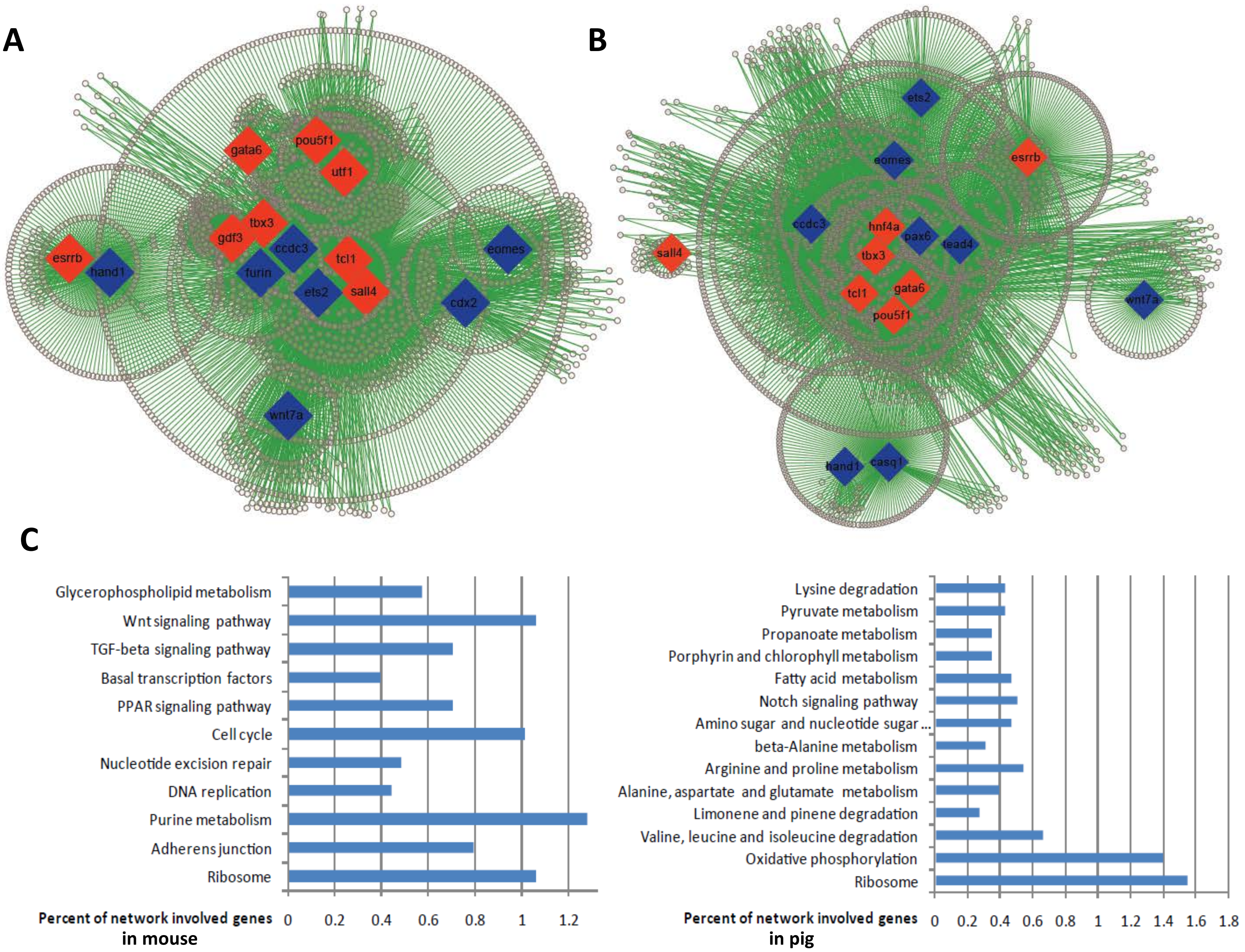
A
gata6
pou5f1
utf1
tbx3
gdf3
ccdc3
esrrb
hand1
furin
tcl1
ets2
sall4
eomes
cdx2
wnt7a
B
ets2
eomes
esrrb
ccdc3
hnf4a
pax6
tead4
sall4
tbx3
tcl1
gata6
pou5f1
wnt7a
hand1
casq1
C
Glycerophospholipid metabolism
Wnt signaling pathway
TGF-beta signaling pathway
Basal transcription factors
PPAR signaling pathway
Cell cycle
Nucleotide excision repair
DNA replication
Purine metabolism
Adherens junction
Ribosome
Percent of network involved genes in mouse
0
0.2
0.4
0.6
0.8
1
1.2
Lysine degradation
Pyruvate metabolism
Propanoate metabolism
Porphyrin and chlorophyll metabolism
Fatty acid metabolism
Notch signaling pathway
Amino sugar and nucleotide sugar ...
beta-Alanine metabolism
Arginine and proline metabolism
Alanine, aspartate and glutamate metabolism
Limonene and pinene degradation
Valine, leucine and isoleucine degradation
Oxidative phosphorylation
Ribosome
Percent of network involved genes in pig
0
0.2
0.4
0.6
0.8
1
1.2
1.4
1.6
1.8
